# Compromised Cerebral Arterial Perfusion, Altered Brain Tissue Integrity, and Cognitive Impairment in Adolescents with Complex Congenital Heart Disease

**DOI:** 10.3390/jcdd11080236

**Published:** 2024-07-29

**Authors:** Nancy A. Pike, Bhaswati Roy, Cristina Cabrera-Mino, Nancy J. Halnon, Alan B. Lewis, Xingfeng Shao, Danny J. J. Wang, Rajesh Kumar

**Affiliations:** 1 Sue & Bill Gross School of Nursing, University of California Irvine, Irvine, CA 92697, USA; 2 The Heart Institute, Children’s Hospital Los Angeles, Los Angeles, CA 90027, USA; 3 Departments of Anesthesiology, University of California Los Angeles, Los Angeles, CA 90095, USA; broy@mednet.ucla.edu; 4 School of Nursing, University of California Los Angeles, Los Angeles, CA 90095, USA; ccabreram@sonnet.ucla.edu; 5 Division of Pediatric Cardiology, Mattel Children’s Hospital UCLA, Los Angeles, CA 90095, USA; nhalnon@mednet.ucla.edu; 6 Division of Pediatric Cardiology, Children’s Hospital Los Angeles, Los Angeles, CA 90027, USA; alewis@chla.usc.edu; 7 Laboratory of FMRI Technology (LOFT), Stevens Neuroimaging and Informatics Institute, Keck School of Medicine, University of Southern California, Los Angeles, CA 90033, USA; xingfeng.shao@loni.usc.edu (X.S.); dannyjwa@usc.edu (D.J.J.W.); 8 Departments of Anesthesiology, Radiological Sciences & Bioengineering, University of California Los Angeles, Los Angeles, CA 90095, USA; rkumar@mednet.ucla.edu

**Keywords:** single ventricle, memory, magnetic resonance imaging, arterial transit time

## Abstract

(1) Introduction: Adolescents with complex congenital heart disease (CCHD) show brain tissue injuries in regions associated with cognitive deficits. Alteration in cerebral arterial perfusion (CAP), as measured by arterial transit time (ATT), may lead to perfusion deficits and potential injury. Our study aims to compare ATT values between CCHD patients and controls and assess the associations between ATT values, MD values, and cognitive scores in adolescents with CCHD. (2) Methods: 37 CCHD subjects, 14–18 years of age, who had undergone surgical palliation and 30 healthy controls completed cognitive testing and brain MRI assessments using a 3.0-Tesla scanner. ATT values and regional brain mean diffusivity [MD] were assessed for the whole brain using diffusion tensor imaging. (3) Results: The mean MoCA values [23.1 ± 4.1 vs. 28.1 ± 2.3; *p* < 0.001] and General Memory Index, with a subscore of WRAML2 [86.8 ± 15.4 vs. 110.3 ± 14.5; *p* < 0.001], showed significant cognitive deficits in CCHD patients compared to controls. The mean global ATT was significantly higher in CCHD patients versus controls (mean ± SD, s, 1.26 ± 0.11 vs. 1.19 ± 0.11, *p* = 0.03), respectively. The partial correlations between ATT values, MD values, and cognitive scores (*p* < 0.005) showed significant associations in areas including the hippocampus, prefrontal cortices, cerebellum, caudate, anterior and mid cingulate, insula, thalamus, and lingual gyrus. (4) Conclusions: Adolescents with CCHD had prolonged ATTs and showed correlation with clinical measurements of cognitive impairment and MRI measurements of brain tissue integrity. This suggests that altered CAP may play a role in brain tissue injury and cognitive impairment after surgical palliation.

## 1. Introduction

Congenital heart disease (CHD) is the most common birth defect in the United States. There are multiple types of CHD, which vary in severity, complexity, and the number of required palliative surgical procedures. Over 50% of children and adolescents with complex CHD (CCHD) have cognitive deficits and show evidence of significant brain tissue injury, particularly in areas that mediate cognitive functions (e.g., visuospatial, language, memory, and executive function) [1,2,3,4,5,6,7]. Injuries associated with these brain regions are linked to worse cognitive outcomes [7,8,9,10,11,12]. Previous studies examining brain tissue integrity in CCHD reported significant brain damage in regions that exert a major influence on cognition (hippocampus, frontal cortex, temporal, and parietal sites) [7,8]. However, the underlying cause of brain injury in these sites in CCHD is unknown.

Cerebral arterial perfusion, which can impact the blood–brain barrier, has been linked to significant brain injury in conditions such as diabetes, epilepsy, multiple sclerosis, hypertension, and Alzheimer’s disease [13,14,15]. Alteration in cerebral arterial perfusion may lead to neural changes and potentially cause brain injury in CCHD patients. Alterations in cerebral arterial perfusion can be related to both structural (i.e., stiffness or congenital abnormalities) and/or functional (i.e., impaired endothelium associated with low cardiac output and/or hypoxemia) differences. Structural changes in the brain can be present in patients with congenital heart defects, affecting the transverse arch or low cardiac output and hypoxemia; these conditions can alter artery perfusion.

Cerebral arterial perfusion and brain tissue injury can be assessed non-invasively by magnetic resonance imaging (MRI), specifically with the use of pseudo-continuous arterial spin labeling (pCASL) procedures [16]. Cerebral arterial perfusion can be measured by the arterial transit time (ATT), which is the time needed for blood to travel from the large arteries to the capillaries. In addition, the brain’s structural integrity can be non-invasively assessed using measures of DTI-based mean diffusivity (MD), which quantifies average water diffusion within tissue; having an MD higher than the control value indicates chronic tissue injury [17,18]. These chronic injuries occur in hypoxic/ischemic conditions and can provide information on the amount of structural brain deficits. However, there are no published reports of cerebral arterial perfusion as measured by ATT values in CCHD patients or any linkages between ATT and MD values regarding cognition. The purpose of this study is to compare ATT values between CCHD patients and age- and gender-matched controls and assess associations between ATT values, MD values, and cognitive scores in adolescents with CCHD.

## 2. Materials and Methods

### 2.1. Study Design, Sample and Setting

This study used a cross-sectional, comparative, and correlational design. Participants were recruited from two tertiary care pediatric cardiology clinics and a local CHD camp. Inclusion criteria were as follows: 14–18 years of age, undergoing surgical palliation for CCHD, and being an English speaker. Exclusion criteria were contraindications for MRI, pregnancy; claustrophobia; prior head injury; brain tumor; cranial surgery; born after less than 37 weeks’ gestation; previous cardiac arrest; extracorporeal membrane oxygenation (ECMO) use; currently listed for heart transplant; and known stroke, genetic syndrome or, severe developmental delays precluding self-report. Age- and sex-matched healthy controls self-reported as having no chronic illnesses, prior stroke, head injury, brain tumor or cranial surgery, seizures, meningitis or central nervous system infection, cardiac arrest, or psychiatric disorders. Recruitment occurred via snowball sampling or word of mouth from the Los Angeles community. Parental permission and assent were obtained from all participants under 18 years of age, and written informed consent was obtained from participants 18 years of age and older before data collection.

### 2.2. Measurements

#### 2.2.1. Montreal Cognitive Assessment (MoCA)

The MoCA is an administered screener used to measure multiple domains of cognitive function (e.g., visuospatial, attention/concentration, executive function, language, delayed memory recall, and naming). Visuospatial and executive function tasks are written items (e.g., three-dimensional cube, Watson Clock Drawing Task, or alternating Trail Making Task Part B), and the remaining are scored based on verbal responses. The total MoCA score ranges from 0 to 30, and a score <26 is considered abnormal. The sensitivity and specificity required to detect mild cognitive impairment are 90% and 87%, respectively [19], and these figures were previously validated in a CHD population, obtaining a Cronbach’s alpha of 0.8 [8].

#### 2.2.2. Wide Range Assessment of Memory and Learning (WRAML2)

The WRAML2 provides a more detailed assessment of cognition in areas of memory (visual/verbal), executive function (working memory), attention/concentration, language, and learning [20]. The WRAML2 takes less than 1 h to administer and is normalized for individuals aged 5–90 years, with mean scores of 100 ± 15 standard deviation. The WRAML2 is composed of 6 subtests with a combined score yielding a General Memory Index. The alpha reliabilities for the core subtests range from 0.85–0.94, specifically, the General Memory Index is 0.93 [20]. The WRAML2-derived General Memory Index score has been widely used to assess memory in adolescents both with and without CHD [7,21].

#### 2.2.3. Demographic and Clinical Data

A demographic form was completed by participants in order to collect information on age, sex, ethnicity, handedness, insurance type, and socioeconomic status (e.g., highest level of education of patient and parent, and household income). Clinical variables were obtained through electronic medical record extraction to verify CHD type, the number of surgical procedures, years from the last surgery, current medications, body mass index, current oxygen saturation level, and first surgery before 30 days.

### 2.3. Data Acquisition

All brain MRI studies were performed on a 3.0-Tesla scanner (Siemens, Magnetom, Prisma-Fit, Erlangen, Germany). All images were reviewed immediately after data acquisition, and if movement or other imaging artifacts were detected, scanning was repeated. The following MRI scans were collected:

#### 2.3.1. Flow-Encoded Arterial Spin Tagging (FEAST) Imaging

FEAST scans were performed for the calculation of ATT using 3D pCASL with a diffusion-prepared gradient and spin-echo readout [repetition time (TR) = 4200 ms; echo time (TE) = 36.04 ms; post-labeling delay = 900 ms; flip angle (FA) = 120°, bandwidth = 3126 Hz/pixel; label offset = 90 mm; labeling duration = 1500 ms; matrix size = 64 × 64; FOV = 240 × 240 mm; 12 slices with slice thickness = 10.0 mm; and 9 measurements with b = 0 and 10 s/mm^2^ with an M0 image for a total scan duration of 3 min] [22,23].

#### 2.3.2. Diffusion Tensor Imaging

DTI was performed using single-shot echo-planar imaging with a twice-refocused spin-echo pulse sequence (TR = 12,200 ms; TE = 87 ms; FA = 90°; bandwidth = 1345 Hz/pixel; matrix size = 128 × 128; FOV = 230 × 230 mm; slice thickness = 1.7 mm; 92 axial slices; no interslice gap; diffusion directions = 30; b = 0; and 800 s/mm^2^). The generalized autocalibrating partially parallel acquisition parallel imaging technique, with an acceleration factor of two, and two separate scans were used for subsequent averaging.

#### 2.3.3. High-Resolution T1-Weighted Imaging

High-resolution T1-weighted images were collected using the MPRAGE (TR = 2200 ms, TE = 2.41 ms, inversion time = 900 ms, FA = 9°), with 320 × 320 matrix size, 230 × 230 mm FOV, 0.9 mm slice thickness, and 192 sagittal slices.

#### 2.3.4. Proton Density and T2-Weighted Imaging

Proton density and T2-weighted images were collected (TR = 10,000 ms, TE1,2 = 12, 124 ms, FA = 130°) using a dual-echo turbo spin-echo pulse sequence in the axial plane, with 256 × 256 matrix size, 230 × 230 mm FOV, 3.5 mm slice thickness, and 50 slices.

### 2.4. MRI Data Processing

High-resolution T1-weighted, PD-, and T2-weighted images were assessed visually for any gross brain pathology, including infarcts, cystic lesions, and tumors, before data processing. FEAST and DTI data were examined for any potential motion or imaging artifacts. Subjects with any gross brain pathology or motion artifacts that could alter assessments were excluded from the analyses.

#### 2.4.1. Calculation of ATT Maps and Global Mean ATT Values

Using FEAST data, ATT maps (unit, s) were generated using the flow-encoding ASL regime by calculating the ratio of diffusion-prepared pCASL signals with a b of 0 and 10 s/mm^2^ [22]. Whole-brain masks were derived from each individual subject. Using ATT maps from each subject, the masked maps of individual subjects were used to calculate mean global ATT values.

#### 2.4.2. Calculation of MD Maps

Using diffusion (b = 800 s/mm^2^)-weighted images, collected from 30 diffusion directions, and non-diffusion (b = 0 s/mm^2^) images, we calculated diffusion tensor matrices for each series with the Diffusion toolkit software (version 0.6.4.1). The diffusion tensor matrices were diagonalized, and principal eigenvalues (λ_1_, λ_2_, and λ_3_) were calculated at each voxel. Mean diffusivity [MD = (λ_1_ + λ_2_ + λ_3_)/3] values were determined at each voxel using principal eigenvalues, and whole-brain MD maps were generated. The whole masked maps of each subject were used to calculate mean global MD values using MD maps.

#### 2.4.3. Realignment, Normalization, and Smoothing of MD and ATT Maps

Both MD maps and b0 images, derived from each DTI series, were realigned and averaged. The averaged MD maps were normalized to the MNI space using averaged b0 images and by implementing a unified segmentation approach [24]. The resulting normalization parameters derived from the b0 images were applied to the corresponding MD maps [25]. The normalized MD maps were smoothed using a Gaussian filter (8 mm), and smoothed MD maps were used for regional tissue injury assessment. Similarly, ATT maps were normalized to the MNI space and smoothed using a Gaussian filter (8 mm).

### 2.5. MRI Data Processing

Power analysis indicated that a total sample size of 70 subjects (37 CHD and 33 controls) could detect moderate-to-large effect sizes of 0.37 at an alpha of 0.05, with a power of 0.85. The demographic data of CCHD and control groups were assessed using an independent samples t-test and the Chi-square method. The global mean MD and ATT values, derived from MD and ATT maps, were compared between CCHD and control groups using ANCOVA, with age and sex as covariates (Bonferroni corrections). All statistical analysis was performed using SPSS V 28.0 software. Whole-brain smoothed ATT and MD maps were correlated voxel-by-voxel with MoCA, WRAML2, and their subscores in CCHD subjects using partial correlations (SPM12; covariates, age, and sex, uncorrected, *p*  <  0.005). Brain clusters showing significant correlations between ATT and MD vs. cognition scores were overlaid onto background images. Region-specific correlation coefficient values were derived using ROI values from specific brain regions that showed significant associations in whole-brain voxel-based correlation analyses. They were then correlated with cognition scores (partial correlations covariates, age and sex, Bonferroni-corrected, *p*  <  0.05).

## 3. Results

### 3.1. Demographic and Characteristics

The demographic and clinical characteristics of CCHD and control participants are summarized in Table 1. There were no statistically significant differences in age, sex, ethnicity, body mass index, handedness, and socioeconomic status between the groups. The most common type of CCHD patient was single-ventricle (62%), had undergone 3 previous surgeries, and was 11 years out from their last surgical procedure, and there were 8 (22%) patients with current oxygen saturations of less than 93%.

### 3.2. Cognitive Scores, MD, and ATT Values between Groups

The mean MoCA [23.1 ± 4.1 vs. 28.1 ± 2.3; *p* < 0.001] and GMI scores [86.8 ± 15.4 vs. 110.3 ± 14.5; *p* < 0.001] show significant cognitive deficits in CCHD patients compared to the controls. ATT values were significantly increased in CCHD patients vs. controls [mean ± SD, s, 1.26 ± 0.11 (n = 30) vs. 1.19 ± 0.11, *p* = 0.03 (n = 28)], respectively, indicating compromised CAI (Table 2).

### 3.3. Partial Correlations between ATT Values and Cognition

**Figure 1:**
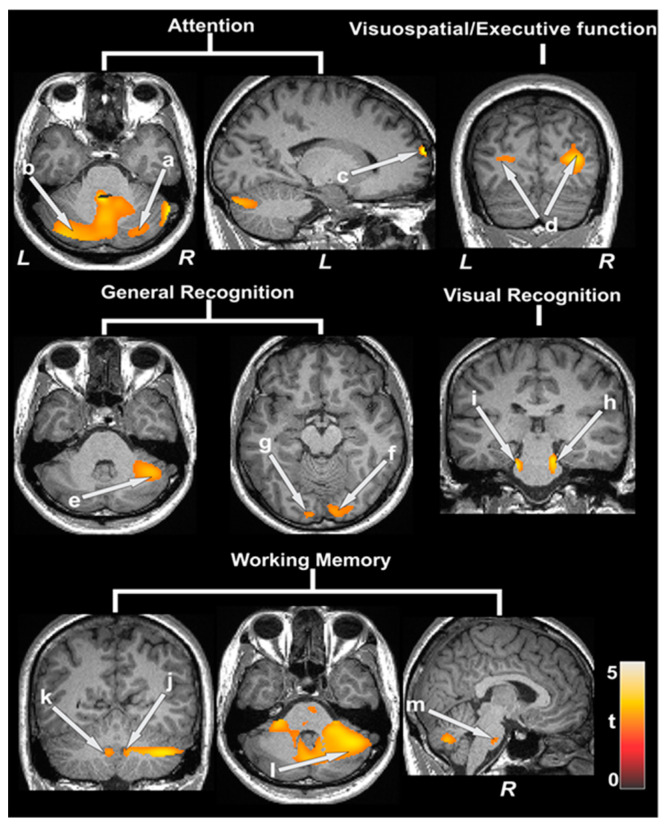
Arterial transit time (ATT) values and cognitive scores. Negative correlations emerged between ATT, MoCA, subscores, WRAML2, and the subscales of CCHD subjects. Negative correlations appeared between ATT and cognition scores at the bilateral cerebellum (a, b), prefrontal cortices (c), bilateral occipital cortices (d) in the MoCA subscale. These were also found for the right cerebellum (e), bilateral occipital (f, g), bilateral mid-brain (h, i), bilateral cerebellar tonsil (j, k), right cerebellum (l), and pons (m) when examining WRAML2 subscores. All images are given according to neurological convention (L = left; R = right). The color bar indicates t-statistic values.

Partial correlations between ATT values and cognition showed significant negative associations in various areas, including the hippocampus, parahippocampal gyrus, amygdala, prefrontal cortices, cerebellum, caudate, putamen, anterior and mid cingulate, insula, thalamus, and lingual gyrus (Table 3). Figure 1 shows the negative correlations between ATT and MoCA, WRAML2 total, and the subscales of CCHD patients (covariates, age, and sex, *p* < 0.005). The dispersion of ATT values are shown in box and whisker plots in Supplementary Figures S1–S4.

### 3.4. Partial Correlations between MD Values and Cognition

**Figure 2:**
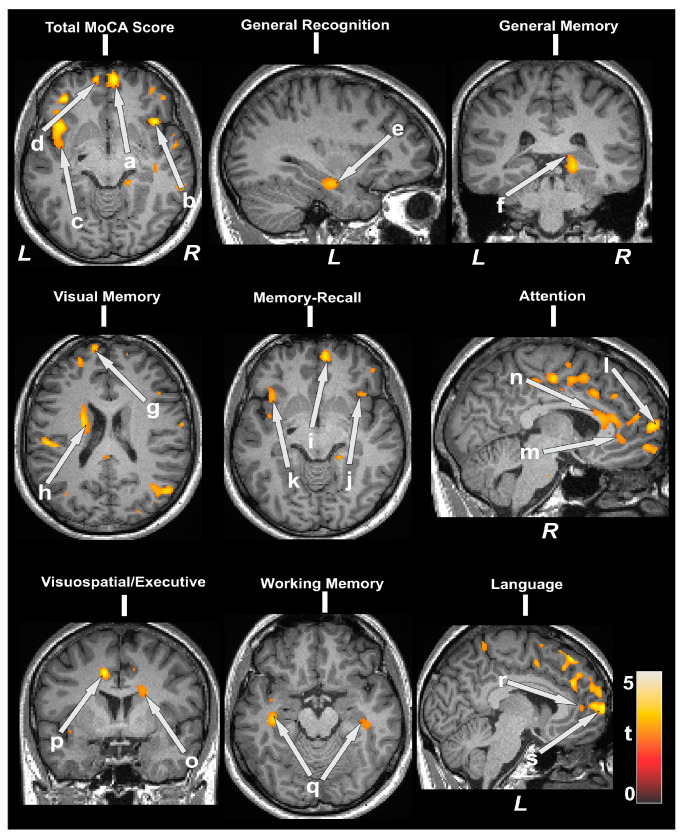
Mean diffusivity (MD) values and cognitive scores. Cognition showed negative associations with MD values in CCHD subjects in several brain sites. Negative correlations appeared between MD and MoCA and subscores at the bilateral prefrontal cortices (a, d, i, l, s), bilateral insula (b, c, j, k), anterior (m, p, r), mid (n) cingulate, and the caudate (o). These were also found for WRAML2 subscales, the hippocampus (e, f, q), prefrontal cortices (g), and the caudate (h, l). All images are presented according to neurological convention (L = left; R = right). The color bar indicates t-statistic values.

Partial correlations between MD values and cognitive scores are shown in Table 4. Negative correlations appeared between MD and MoCA subscores at the bilateral prefrontal cortices, bilateral insula, anterior, and mid cingulate, and the caudate. These were also found for WRAML2 subscales, hippocampus, prefrontal cortices, and the caudate. Figure 2 shows negative correlations between MD values in CCHD in multiple brain regions (covariates, age, and sex, *p* < 0.005). The dispersion of MD values is shown in box and whisker plots in Supplementary Figures S5–S7.

### 3.5. Structural Brain MRI Findings

There were abnormal brain MRI findings in the CCHD group for 12 out of 37 (32%) patients compared to 2 out of 33 (5%) in the control group (Table 5). The cerebral lesions and alterations detected were white matter changes, old infarctions/strokes, Chiari I malformations, and periventricular volume losses. Incidental developmental abnormalities were seen in both groups. 

## 4. Discussions

Our findings in adolescents with CCHD suggest that compromised cerebral arterial perfusion may play a key role in cognitive deficits. This is the first study to measure ATT values using pCASL techniques in a CCHD cohort. Increased ATT values were identified in select brain regions that underlie multiple cognitive and social-emotional functions (e.g., hippocampus, prefrontal cortices, cerebellum, caudate, anterior/mid cingulate, insula, thalamus, and lingual gyrus) in adolescents with CCHD.

Two previous studies examined CBF using ASL techniques but did not measure ATT values [26,27]. Overall, one study into preoperative neonates with CCHD found reduced global and regional CBF [27]. However, younger children with single-ventricle heart disease were at high risk of developing aortic to pulmonary collaterals, which contribute to reduced CBF [28,29], and progressive hypoxemia, which can potentially impact cognitive function.

Another study, performed using a heterogeneous cohort of children and adolescents with CCHD, identified age-related declines in CBF compared to controls in the brain regions of the prefrontal, insula, cingulate, precuneus, and parietal, and these were found to mediate cognitive performance scores [26]. Reductions in CBF and increased ATT values in the middle and posterior cingulate and frontal cortices were negatively correlated with cognitive impairment in the population with Alzheimer’s disease [30,31]. A Danish study of over 10,000 adult CHD patients showed a 60% increase in the risk of early dementia compared to the general population [32]. The risk was higher in those with single-ventricle physiologies and sedentary lifestyles [32]. Our adolescent cohort of single ventricle and TOF patients showed increased ATT and MD values compared to controls in similar brain regions of the middle to posterior cingulate and prefrontal cortices. A preliminary CCHD group analysis identified worse ATT and MD values in single-ventricle participants compared to TOF patients. Our study demonstrates the vulnerability of the cerebral vascular system in adolescents with CCHD, which may persist into adulthood.

ATT is a crucial parameter in understanding blood flow dynamics and the accurate measurement of this parameter can lead to it use as a biomarker or for effective treatment planning in conditions involving compromised blood flow (e.g., stroke, hepatic encephalopathy with cirrhosis, seizures, and neurodegeneration) [33,34,35]. In stroke management, delayed ATT can indicate the presence of collateral blood flow, which can influence treatment decisions, such as the administration of thrombolytic therapy or mechanical thrombectomy [35]. Further investigation is needed to assess the usefulness of ATT values as non-invasive surveillance biomarkers for early neurodegeneration in CCHD.

ATT can be examined non-invasively with MRI techniques and is suitable for children since it does not involve the use of invasive venous cannulation or radiation. Currently, there are no comparative ATT studies available in the pediatric CHD literature. Adult ATT values are not comparable due to age-related changes in cerebral perfusion. One study examines ATT values in normally developing children between 7 and 17 years of age. Using FEAST, we found that the reported mean ATT was 1.5 s in a cohort of 18 children, with a trend of increased values with aging, indicative of worsening cerebral perfusion [36]. However, the authors acknowledge the potential for overestimations of ATT values due to the use of venous instead of arterial blood T1 values, and the use of 3.0-Tesla MRI compared to 1.5-Tesla. A stronger magnetic field has an advantage in ASL, as it provides an increased signal-to-noise ratio and prolonged tracer half-life due to increases in T1 values [36]. In the current study, our healthy controls, of a similar age range, had a mean ATT value of 1.2 compared to 1.5 s in the referenced study. This could be due to various procedural aspects of data acquisition, such as the use of diffusion-prepared 3D pCASL in our study instead of a diffusion-weighted 2D pCASL sequence. The sequential 2D slice acquisitions in the study of Jain et al. could result in a longer ATT as the top slices were acquired almost 1 s later than the bottom slices. Longitudinal surveillance of ATT values in single-ventricle patients could be useful in identifying trends in aging and targeted time points for early intervention.

Interestingly, our study identified the cerebellum as a brain region with a significant relationship between ATT values; MD values; the cognitive scores associated with attention, visual–spatial, visual, and general recognition; and working memory functions. The cerebellum’s role in motor function is well recognized, but the nature of its concurrent role in cognitive function remains unclear and a matter of debate [37]. The cerebellum also appears to have less capability to remodel [37]. Thus, researchers have reported that cerebellar disturbances in early life are linked to an increased risk of developing autism spectrum disorder [38]. A large case-control study confirmed that children born with CHD had a 33% increased likelihood of being diagnosed with autism spectrum disorder even after controlling for other factors known to elevate the risk of autism, including genetic syndromes, prematurity, and neonatal complications such as epilepsy or insufficient oxygen at birth [39]. The risks were the highest among children with less critical forms of CHD (e.g., atrial septal defects, ventricular septal defects), or more CCHD [39]. With most cardiac surgeries performed in the first year of life, it is plausible that young children with CCHD are at higher risk of cerebellar injury and potentially autism. Though this study excluded autism and genetic syndromes with known cognitive impairments, we found an association between ATT values and executive function tasks in the cerebellum. This study provides support to the premise that the cerebellum forms “closed loop” connections with neocortical brain regions. This includes supporting the notion that the prefrontal cortex plays a role in higher cognitive function [40].

Several intraoperative variables can affect the perfusion of the developing brain, including the use of a cardiopulmonary bypass, aortic cross clamps, deep hypothermic circulatory arrest (DHCA) times, the cooling duration and degree, and regional (antegrade) cerebral perfusion [41]. Many researchers hoped that the use of regional cerebral perfusion would lessen the adverse effects of DHCA, but the benefits were not demonstrated in numerous studies [41,42,43]. The type of intraoperative management strategy contributed less variance to cognitive outcomes than patient-specific and preoperative factors, perioperative hemodynamic instability, and postoperative morbidities [44,45,46]. Unfortunately, in this study, we were unable to retrieve many participants’ intraoperative data to assess their effects on cognitive impairment.

Our findings suggest that the effect of cerebral arterial perfusion normalization on cognitive outcomes should be studied in the CCHD population and may represent a promising target for early interventions to improve cognitive function.

### Study Limitations

This study should be viewed in a way that considers some of its limitations. Despite the small sample size, significant differences in cognitive scores and ATT values emerged between the groups. The small sample precluded our ability to examine the data based on CCHD type or identify predictors. The CCHD sample may reflect a group with better health within chronic condition due to the extensive exclusion criteria (gestation < 37 weeks, no previous stroke, extracorporeal membrane oxygenation use, cardiac arrest, and pacemaker use), and the results may not be generalizable to all the CCHD population. Lastly, we examined adolescents with CCHD at one time point to assess cumulative injury and were unable to determine the timing of and mechanism by which alterations ATT values started and the extent of brain tissue injury due to the condition.

## 5. Conclusions

Adolescents with CCHD have compromised cerebral arterial perfusion, which indicates cerebral hypoperfusion, and there are significant associations between cerebral arterial perfusion and brain tissue integrity, with implications for cognitive function. Negative correlations were predominantly seen between ATT values, MD values, and cognition scores in multiple cortical and subcortical brain regions (prefrontal, hippocampus, amygdala, prefrontal cortices, cerebellum, insula, cingulate, and the caudate) that mediate cognitive function. These findings may result from innate factors, the complications of surgery, or reduced ventricle function, altering physiology and circulation.

## Figures and Tables

**Table 1:** Sample characteristics between CCHD and control groups.

Characteristic	CCHD Group [n = 37]	Control Group [n = 33]	*p*-Value
	Mean [SD] or n [%]		
Age [years]	15.8 [1.37]	15.8 [1.21]	0.881
Gender [female]	19 [51%]	19 [58%]	0.638
Ethnicity			0.75
White	16 [43%]	13 [39%]	
Hispanic	17 [46%]	15 [45%]	
Other	4 [11%]	5 [15%]	
Body mass index (kg/m^2^)	23 [6.3]	24.4 [4.8]	0.467
Handedness [Right]	30 [81%]	30 [91%]	0.411
Household income [highest]			0.233
<30,000	11 [30%]	5 [15%]	
30,000–50,000	5 [13.5%]	1 [3%]	
50,001–80,000	7 [19%]	7 [21%]	
81,001–100,000	2 [6%]	6 [18%]	
>100,000	9 [24%]	13 [40%]	
Unsure	3 [8%]	1 [3%]	
Maternal education [highest]			0.725
<High school	10 [27%]	7 [21%]	
High school graduate	11 [30%]	9 [27%]	
College or university degree	10 [27%]	10 [31%]	
Graduate degree	6 [16%]	7 [21%]	
CCHD type		N/A	N/A
Tetralogy of Fallot	14 [38%]		
Single-ventricle/Fontan	23 [62%]		
Number of surgeries	3 [0.7]	N/A	N/A
Number of years since last surgery	11 [5.1]	N/A	N/A
Oxygen saturation <93%	8 [22%]	N/A	N/A

Abbreviations: complex congenital heart disease (CCHD), not applicable (N/A)

**Table 2:** Cognitive scores and arterial transit times (ATT) between CCHD and control groups.

Variables	CCHD Group [n = 37]	Control Group [n = 33]	*p* Value
	Mean [SD]		
MoCA, Total	23.1 [4.1]	28.1 [2.3]	<0.001
Visuospatial/executive function	3.59 [1.2]	4.48 [0.9]	0.002
Naming	2.95 [0.2]	3.00 [0.0]	0.18
Attention	4.19 [1.4]	5.45 [1.0]	<0.001
Language	1.68 [1.0]	2.64 [0.6]	<0.001
Abstraction	0.95 [0.7]	1.88 [0.3]	<0.001
Delayed recall	2.81 [1.4]	3.91 [1.1]	<0.001
WRAML2, General Memory Index	86.89 [15.4]	110.3 [14.5]	<0.001
Verbal memory	87.14 [13.3]	101.21 [13.1]	<0.001
Visual memory	102.41 [13.3]	118.94 [14.1]	<0.001
Attention/concentration	82.70 [15.9]	102.73 [13.3]	<0.001
WRAML2, General Recognition Index	95.67 [13.6]	108.70 [13.6]	<0.001
Working memory	87.59 [17.5]	105.36 [19.1]	<0.001
Verbal recognition	92.62 [13.4]	100.42 [17.4]	0.038
Visual recognition	99.94 [13.9]	108.03 [20.7]	0.06
Arterial transit time [ATT] [s]	1.3 [0.13]	1.22 [0.13]	0.02
Mean diffusivity values	0.98 [0.06]	0.94 [0.06]	0.006

Abbreviations: Montreal Cognitive Assessment [MoCA]; Wide Range Assessment of Memory and Learning 2nd Edition [WRAML2].

**Table 3:** Partial correlations arterial transit time (ATT) values and cognitive scores.

Cognitive Variables vs. ATT	Brain Regions	*r* (*p*-Value)
MoCA vs. ATT	Right cerebellum	−0.51 (0.006)
	Left anterior cingulate	−0.50 (0.007)
	Right anterior insula	−0.51 (0.006)
	Left prefrontal cortices	−0.52 (0.004)
Abstraction vs. ATT	Right thalamus	−0.59 (0.001)
Attention vs. ATT	Left prefrontal	−0.51 (0.006)
Language vs. ATT	Right cerebellum	−0.53 (0.004)
	Left anterior cingulate	−0.50 (0.007)
	Right lingual gyrus	−0.50 (0.007)
	Left mid cingulate	−0.49 (0.008)
Delayed recall vs. ATT	Left prefrontal cortices	−0.49 (0.008)
Visuospatial vs. ATT	Left amygdala	−0.52 (0.005)
	Left hippocampus	−0.54 (0.003)
	Left parahippocampal gyrus	−0.51 (0.006)
	Right lingual gyrus	−0.51 (0.005)
	Left lingual gyrus	−0.53 (0.004)
General memory vs. ATT	Left caudate	−0.50 (0.007)
	Right anterior insula	−0.54 (0.003)
	Left prefrontal cortices	−0.53 (0.004)
General recognition vs. ATT	Left anterior cingulate	−0.50 (0.007)
	Left mid cingulate	−0.49 (0.008)
	Right prefrontal cortices	−0.55 (0.002)
Verbal recognition vs. ATT	Left caudate	−0.52 (0.005)
	Right anterior insula	−0.56 (0.002)
	Right posterior insula	−0.51 (0.006)
	Right prefrontal cortices	−0.55 (0.003)
Verbal memory vs. ATT	Right anterior insula	−0.49 (0.006)
Visual memory vs. ATT	Right prefrontal cortices	−0.51 (0.006)
Working memory vs. ATT	Left prefrontal cortices	−0.50 (0.006)

Abbreviations: Montreal Cognitive Assessment [MoCA]; *r* = correlation coefficient (covariates: age and sex).

**Table 4:** Partial correlation mean diffusivity (MD) values and cognitive scores.

Cognitive Variables vs. MD	Brain Regions	*r* (*p*-Value)
MoCA total vs. MD	Right prefrontal	−0.47 (0.004)
	Left prefrontal	−0.52 (0.001)
	Right insula	−0.52 (0.001)
	Left insula	−0.55 (<0.001)
	Right anterior cingulate	−0.55 (<0.001)
	Left anterior cingulate	−0.54 (<0.001)
	Right mid cingulate	−0.51 (0.002)
	Left mid cingulate	−0.54 (<0.001)
Visuospatial/executive function vs. MD	Left prefrontal	−0.48 (0.004)
	Left insula	−0.50 (0.002)
	Right anterior cingulate	−0.53 (0.001)
	Left anterior cingulate	−0.50 (0.002)
	Right mid cingulate	−0.49 (0.003)
	Left mid cingulate	−0.51 (0.002)
	Caudate	−0.48 (0.004)
Attention vs. MD	Right prefrontal	−0.48 (0.004)
	Left prefrontal	−0.53 (0.001)
	Right insula	−0.48 (0.004)
	Left insula	−0.52 (0.002)
	Right anterior cingulate	−0.54 (<0.001)
	Left anterior cingulate	−0.55 (<0.001)
	Right mid cingulate	−0.51 (0.002)
	Left mid cingulate	−0.54 (<0.001)
Delayed memory recall vs. MD	Right prefrontal	−0.50 (0.002)
	Right insula	−0.47 (0.004)
	Left insula	−0.48 (0.003)
Language vs. MD	Right prefrontal	−0.50 (0.002)
	Left prefrontal	−0.57 (<0.001)
	Left insula	−0.45 (0.006)
	Left anterior cingulate	−0.49 (0.003)
	Right mid cingulate	−0.49 (0.003)
	Left mid cingulate	−0.46 (0.006)
General memory vs. MD	Hippocampus	−0.48 (0.003)
	Prefrontal	−0.43 (0.01)
	Caudate	−0.44 (0.008)
General recognition vs. MD	Hippocampus	−0.52 (0.002)
Working memory vs. MD	Hippocampus	−0.44 (0.008)
Visual memory vs. MD	Hippocampus	−0.48 (0.004)
	Prefrontal	−0.49 (0.003)
	Caudate	−0.46 (0.005)

Abbreviations: Montreal Cognitive Assessment (MoCA); *r* = correlation coefficient (covariates: age and sex).

**Table 5:** Structural Brain MRI findings in CCHD and Controls.

Brain MRI Findings	CCHD [n = 37]	Controls [n = 33]
Any abnormality *n* [%]	12 [32%]	2 [5%]
Focal or multifocal abnormality		
Focal infarction or atrophy	7 [22%]	0 [0%]
Developmental abnormality Minor ^a^	4 [14%]	

^a^ Minor malformations include Rathke’s cleft cysts, pituitary adenoma, and benign perivascular region cysts.

## Data Availability

The authors declare their willingness to share de-identified data that support the findings of this study and are available upon request from the corresponding authors; data-use agreements will be needed to be completed and approved to share data by the University of California, Los Angeles.

## Supplementary Materials

The following supporting information can be downloaded at: https://www.mdpi.com/article/10.3390/jcdd11080236/s1. The dispersion of ATT values are shown in box and whisker’s plots the in Supplementary Figures S1–S4 per the outline in Table 3. The dispersion of MD values are shown in box and whisker’s plots in Supplementary Figures S5–S7 per the outline in Table 4.
