# Supplementary figures and images for: Compromised Cerebral Arterial Perfusion, Altered Brain Tissue Integrity, and Cognitive Impairment in Adolescents with Complex Congenital Heart Disease
Source: J Cardiovasc Dev Dis. 2024 Jul 29;11(8):236. doi: 10.3390/jcdd11080236 (PMC11354402; doi:10.3390/jcdd11080236)

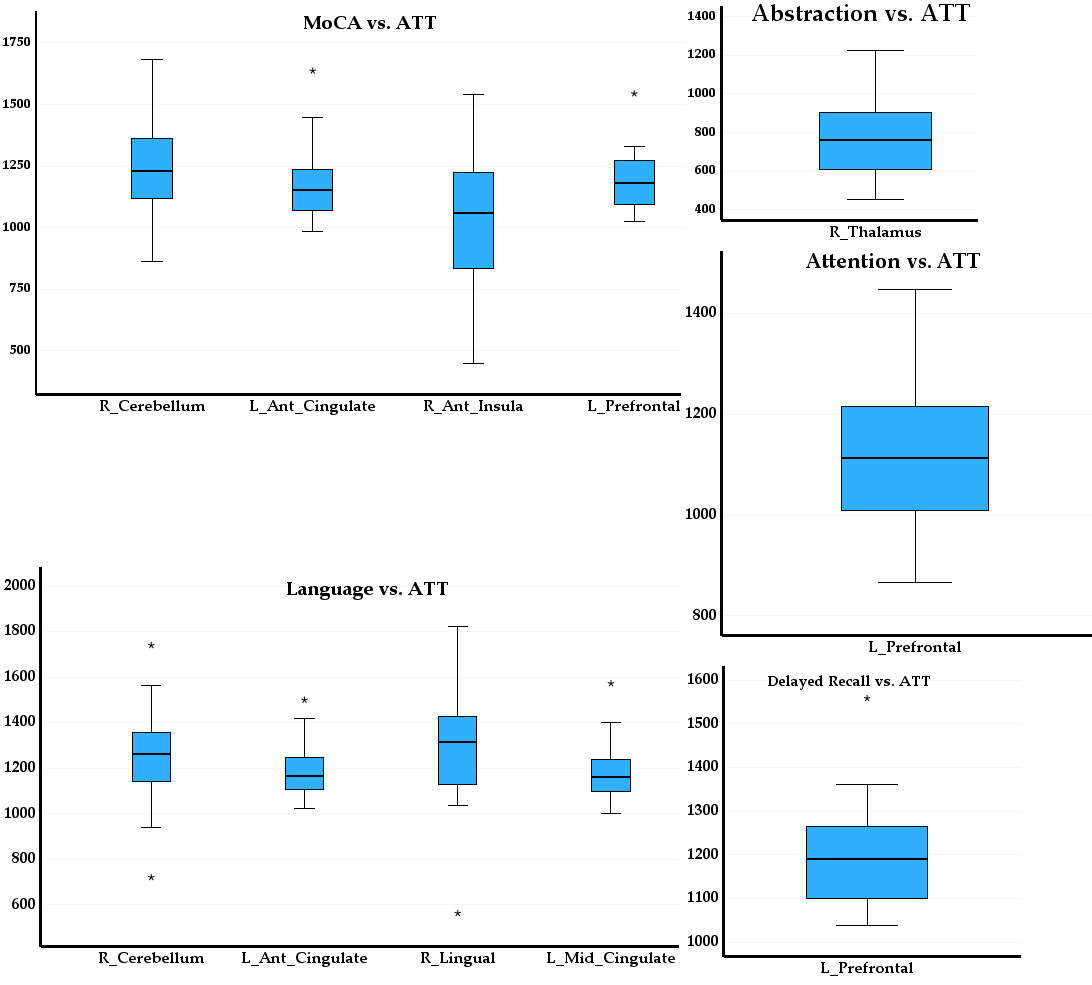

Supplement: Supplementary file 1 [file jcdd-11-00236-s001.zip › Supp Fig-1.tif]

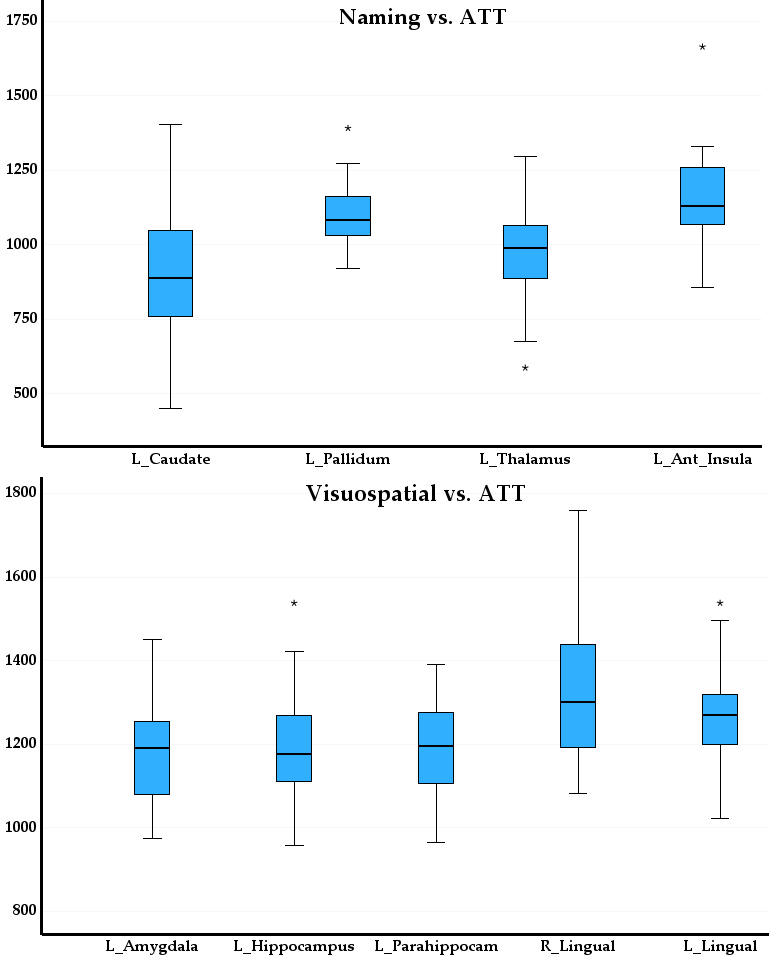

Supplement: Supplementary file 1 [file jcdd-11-00236-s001.zip › Supp Fig-2.tif]

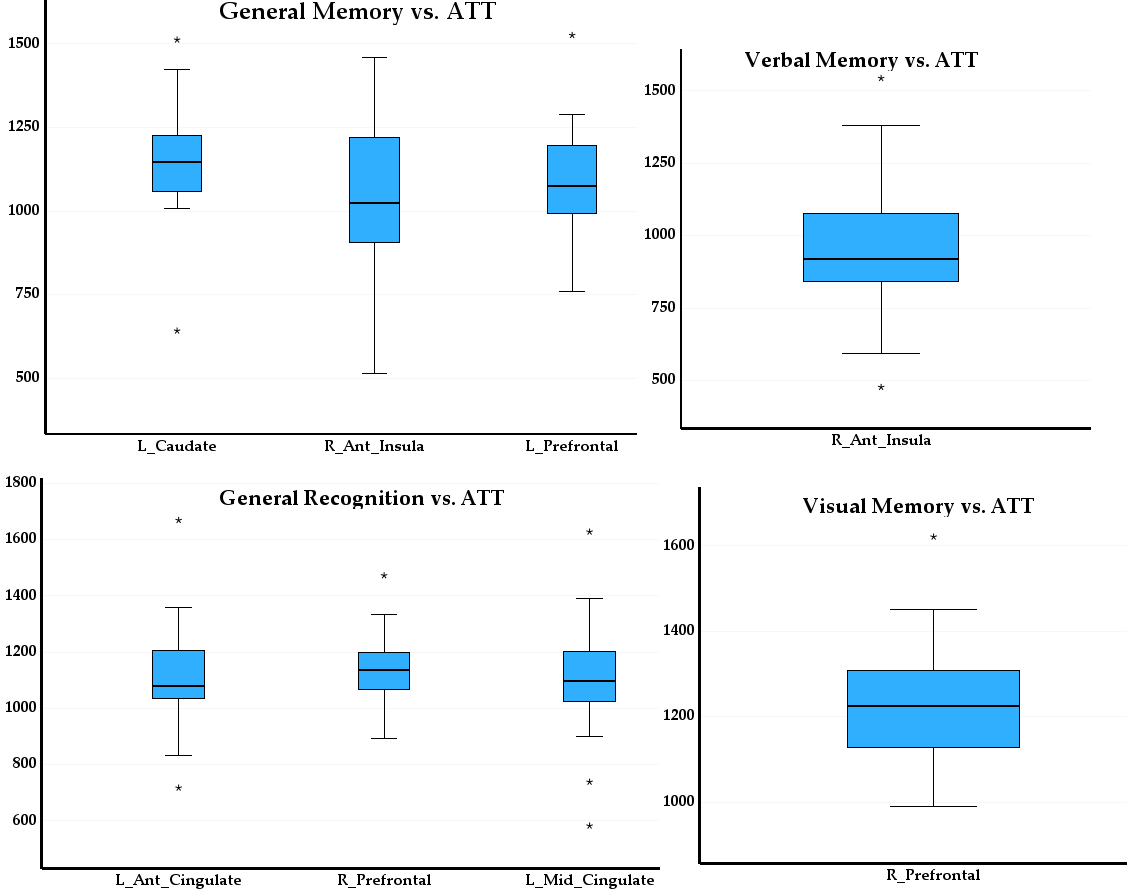

Supplement: Supplementary file 1 [file jcdd-11-00236-s001.zip › Supp Fig-3.tif]

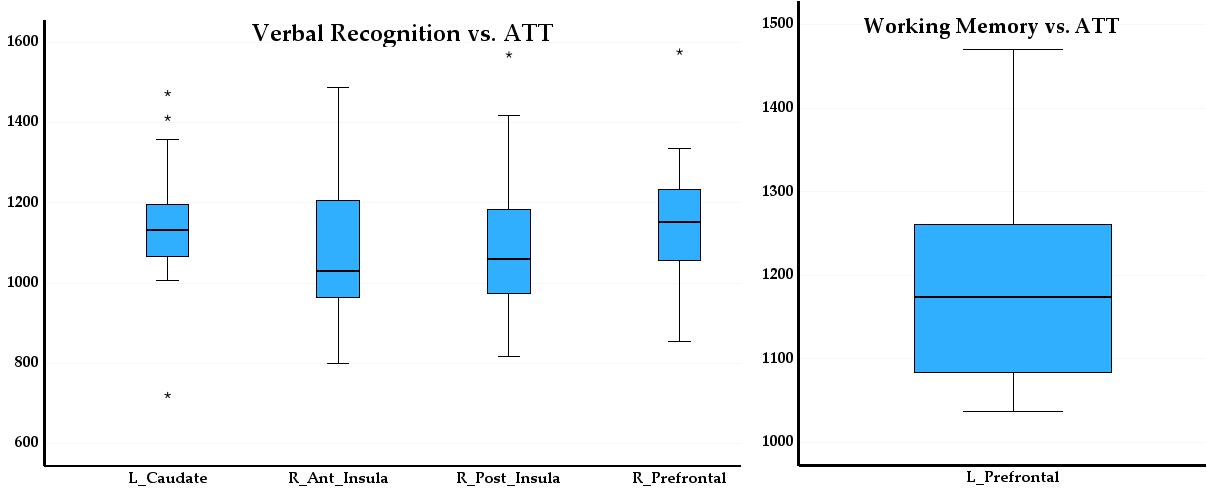

Supplement: Supplementary file 1 [file jcdd-11-00236-s001.zip › Supp Fig-4.tif]

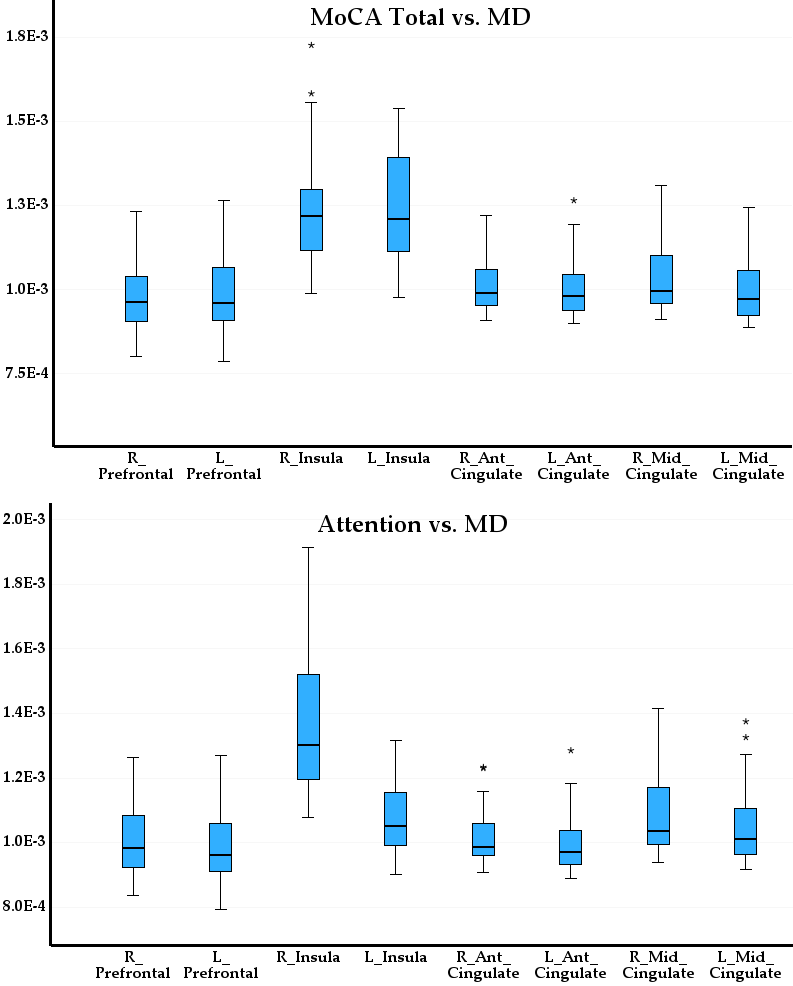

Supplement: Supplementary file 1 [file jcdd-11-00236-s001.zip › Supp Fig-5.tif]

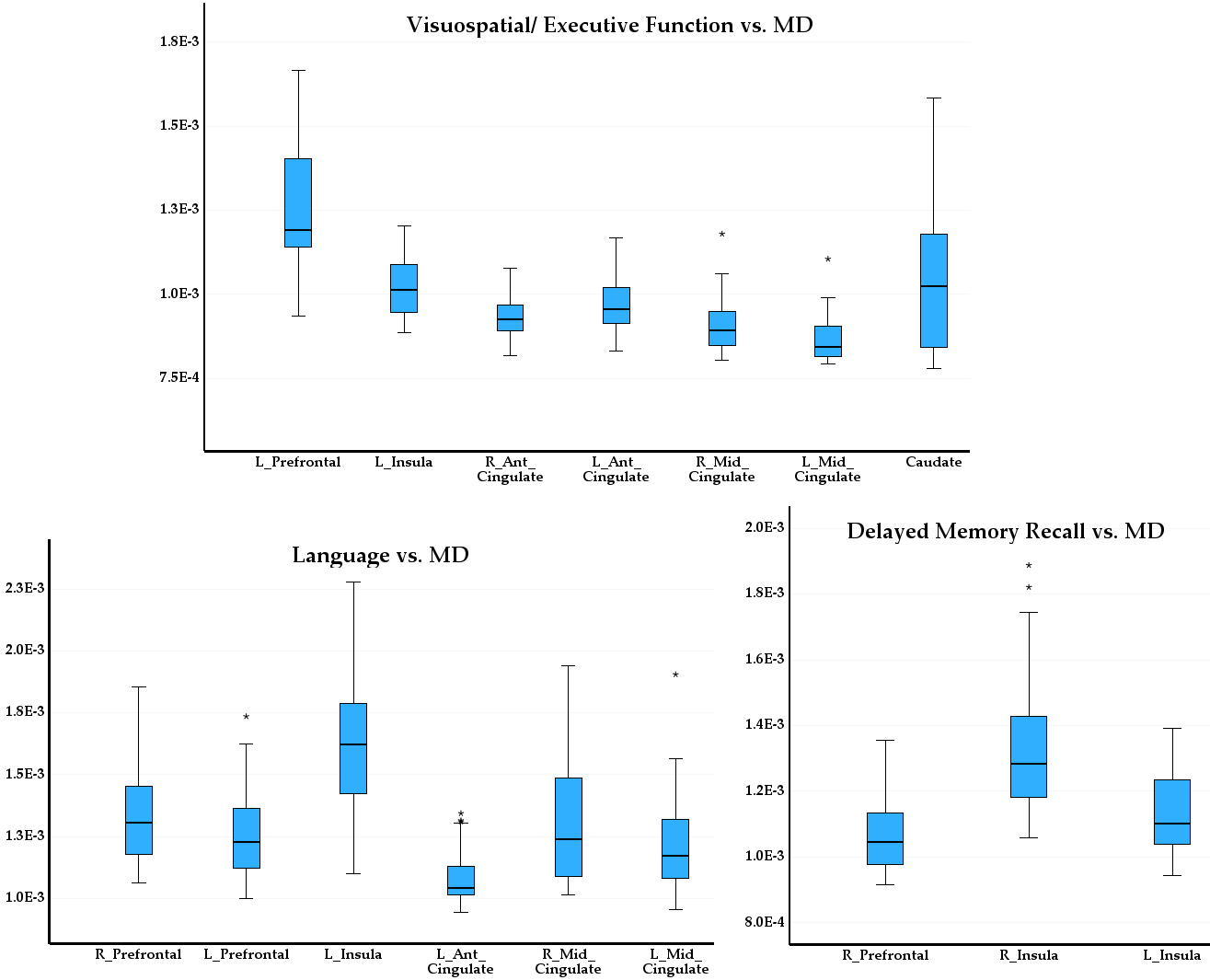

Supplement: Supplementary file 1 [file jcdd-11-00236-s001.zip › Supp Fig-6.tif]

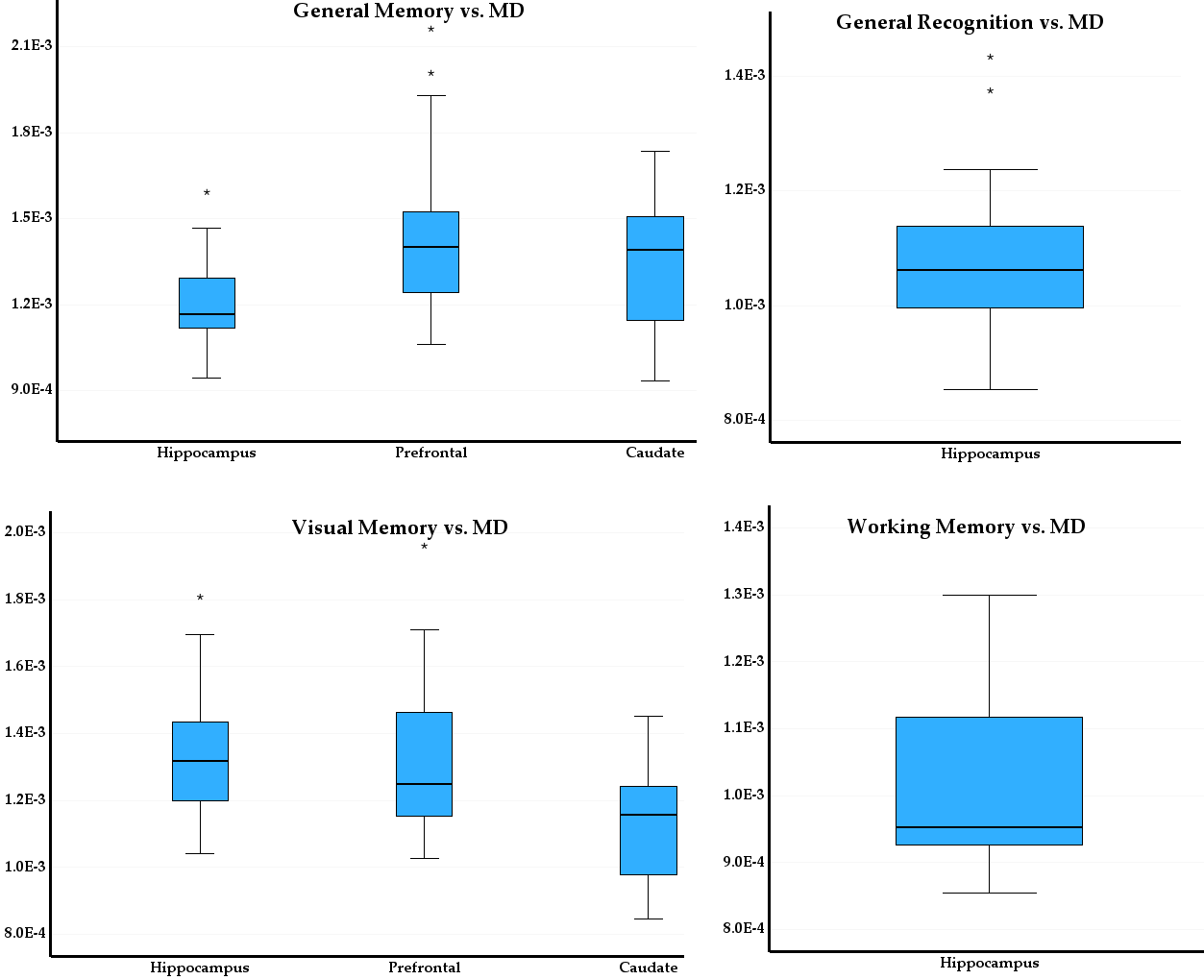

Supplement: Supplementary file 1 [file jcdd-11-00236-s001.zip › Supp Fig-7.tif]
